# Identification and enzymatic characterization of acid phosphatase from *Burkholderia gladioli*

**DOI:** 10.1186/1756-0500-7-221

**Published:** 2014-04-09

**Authors:** Tiago Henrique Rombola, Eliamar Aparecida Nascimbem Pedrinho, Eliana Gertrudes de Macedo Lemos, Adriano Marques Gonçalves, Luiz Flávio José dos Santos, João Martins Pizauro

**Affiliations:** 1 Faculdade de Ciências Agrárias e Veterinárias (FCAV), UNESP – Univ Estadual Paulista, Câmpus de Jaboticabal, Departamento de Tecnologia, Laboratório de Enzimologia Aplicada, Jaboticabal, SP, Brazil; 2 Instituto de Química (IQ), Univ Estadual Paulista, Câmpus de Araraquara, Araraquara, SP, Brazil

**Keywords:** Phosphohydrolase, Inhibition, Phosphate, *P*-Nitrophenylphosphate, Solubilization

## Abstract

**Background:**

The genus *Burkholderia* is widespread in diverse ecological niches, the majority of known species are soil bacteria that exhibit different types of non-pathogenic interactions with plants. *Burkholderia* species are versatile organisms that solubilize insoluble minerals through the production of organic acids, which increase the availability of nutrients for the plant. Therefore these bacteria are promising candidates for biotechnological applications.

**Results:**

*Burkholderia sp.* (R 3.25 isolate) was isolated from agricultural soil in Ponta Grossa-PR-Brazil and identified through analysis of the 16S rDNA as a strain classified as *Burkholderia gladioli*. The expression of membrane-bound acid phosphatase (MBAcP) was strictly regulated with optimal expression at a concentration of phosphorus 5 mM. The apparent optimum pH for the hydrolysis of *p*-nitrophenylphosphate (PNPP) was 6.0. The hydrolysis of PNPP by the enzyme exhibited a hyperbolic relationship with increasing concentration of substrate and no inhibition by excess of substrate was observed. Kinetic data revealed that the hydrolysis of PNPP exhibited cooperative kinetics with n = 1.3, V_m_ = 113.5 U/mg and K_0.5_ = 65 μM. The PNPPase activity was inhibited by vanadate, *p*-hydroxymercuribenzoate, arsenate and phosphate, however the activity was not inhibited by calcium, levamisole, sodium tartrate, EDTA, zinc, magnesium, cobalt, ouabain, oligomycin or pantoprazol.

**Conclusion:**

The synthesis of membrane-bound non-specific acid phosphatase, strictly regulated by phosphate, and its properties suggest that this bacterium has a potential biotechnological application to solubilize phosphate in soils with low levels of this element, for specific crops.

## Background

The genus *Burkholderia* is widespread in diverse ecological niches; however, the majority of known species are soil bacteria that exhibit different types of non-pathogenic interactions with plants [1,2]. Following the pioneering work of Yabuuchi *et al.*[3], which described the *Burkholderia* genus, several investigators have studied *Burkholderia* species that are phylogenetically distant from the *Burkholderia cepacia complex* (Bcc species), which are promising candidates for biotechnological applications [4,2], although their environmental distribution and relevant characteristics for agro-biotechnological applications are not well known.

*Burkholderia* species are versatile organisms that solubilize insoluble minerals through the production of organic acids, which increase the availability of nutrients for the plant [5-7]. Interactions between plant roots and mineral phosphate solubilizing (MPS) microorganisms can play an important role in phosphorus nutrition and growth of most plants, microorganisms and crop production. As far as we know, the present report is the first systematic study to show that the membrane-bound acid phosphatase expressed by *Burkholderia* is strictly regulated by phosphorus. In addition, little is known about the enzyme’s potential applications to improve plant growth by association with the bacteria.

Phosphorus is an essential nutrient that is required in large amounts to maintain levels of key cell molecules, including ATP, nucleic acids, and phospholipids; phosphorus is also a pivotal mediator in the regulation of many metabolic processes, such as energy transfer, protein activation, regulation of enzyme activities, gene activity control, as so in carbon and amino acid metabolic processes [8].

The uptake of nutrients from different natural environments depends on the secretion of an enormous variety of hydrolytic enzymes, which demonstrate catalytic activity that is specific for the cleavage of a particular substrate. This uptake process is tightly regulated and contains a variety of biochemical reactions that involve the acquisition, storage, and release of enzymes [9]. The study of these processes may provide new insights for the elucidation of gene expression that controls not only the synthesis but also the secretion of enzymes by eukaryotic cells in response to environmental factors, such as pH and levels of carbon, nitrogen, sulfur and phosphorus [10,11].

In this paper, we report the expression and kinetic characterization of a membrane-bound acid phosphatase produced by *Burkholderia gladioli* that was isolated from the rhizosphere of *Zea mays*, which was collected from an agricultural soil in Ponta Grossa-PR-Brazil.

## Methods

### Isolation and identification of *Burkholderia sp*

The isolation of *Burkholderia* sp. bacteria from surface-sterilized roots of *Zea mays*, which were collected from agricultural soil in Ponta Grossa-PR-Brazil, was described by Pedrinho *et al.*[12], and the bacteria was identified through partial 16S rRNA gene sequencing, using the specific oligonucleotides fD1 and rD1 [13].

The partial sequencing of the 16S rRNA gene was performed by the use of 1,0 μL of DNA Sequencing-Big Dye Terminator Cycle Sequencing-Ready ABI Prism (Version 3); 3.2 pmols of fD1/rD1 oligonucleotide, 60 ng of DNA, 4.6 μL of buffer (400 mM Tris–HCl, pH 9; 10 mM (MgCl2); and mili-Q (Millipore) H2O for a 10 mL volume.

The amplicons were sequenced using the model ABI 3100 capillary sequencer (Applied Biosystems, Foster City, CA, USA). The fasta sequence was analyzed by comparison using a local tool BLASTN [14] from NCBI (National Center of Biotechnology Information) and classified by RDP (Ribosomal Database Project).

The evolutionary history was inferred using the Neighbor-Joining method [15]. The optimal tree with the sum of branch length = 0.19803035 is shown. The percentage of replicate trees in which the associated taxa clustered together in the bootstrap test (1000 replicates) are shown next to the branches [16]. The tree is drawn to scale, with branch lengths in the same units as those of the evolutionary distances used to infer the phylogenetic tree. The evolutionary distances were computed using the p-distance method [17] and are in the units of the number of base differences per site. The analysis involved 11 nucleotide sequences. Codon positions included were 1st + 2nd + 3rd + Noncoding. All positions containing gaps and missing data were eliminated. There were a total of 733 positions in the final dataset. Evolutionary analyses were conducted in MEGA5 [18]. The 16S rDNA sequence obtained is registered at the International Gene Bank (GenBank), having the access number: JN 700991.

### Growth conditions and membrane-bound enzyme isolation

*Burkholderia* sp. was grown in a liquid medium containing 2% glucose, 0.1% magnesium chloride, 0.025% magnesium sulfate, 0.1% ammonium sulfate, 0.02% potassium chloride, and with or without potassium phosphate. The liquid growth medium, prepared in 250-ml conical flasks, was incubated for 72 h at 30°C under constant rotary shaking at 140 rpm. Actively growing cells were collected by centrifugation, washed twice with 50 mM sodium acetate buffer at pH 6.0, resuspended in 8 mL of the same buffer and then disrupted by sonication at 50 microtips/second with cycles of 30 seconds with a Branson Sonifier model 250. The integral cells were removed by centrifugation at 5,000 g for 15 min. The supernatant was subjected to a two-step differential centrifugation, first at 12,000 g and then for 1 h at 160,000 g to obtain soluble proteins and membrane bound enzyme. The pellet, which corresponds to the membrane-bound enzyme, was resuspended in the same buffer. Aliquots (1.0 ml) were frozen in liquid nitrogen and stored at -20°C without appreciable loss of activity when stored for less than 2 months.

### Enzymatic activity measurements

Acid phosphatase activity was determined discontinuously at 37°C, 50 mM acetate buffer, pH 6.0, through the formation of *p*-nitrophenolate (ϵ = 17600 M^-1^ cm^-1^, pH 13) at 410 nm from the hydrolysis of 1 mM *p*-nitrophenylphosphate (SIGMA®). The enzymatic reaction was initiated by the addition of the enzyme extract to the reaction medium, interrupted by adding 1 ml of 1 M NaOH, and the absorbance was determined at 410 nm.

The determinations were performed in triplicates and the initial velocities remained constant during the incubation time to ensure that substrate hydrolysis was inferior to 5%. In each determination standards were included to estimate the non-enzymatic hydrolysis of substrate.

A unit of enzyme activity was defined and expressed as the amount of enzyme that releases one nmol of *p*-nitrophenolate per minute, per milligram of protein present in the enzymatic extract, under test conditions.

### Thermal inactivation of membrane-bound acid phosphatase

Samples of membrane-bound enzyme in 50 mM acetate buffer at pH 6.0 were incubated in a water bath at different temperatures for variable periods of time. Immediately after the water bath treatment, samples were chilled in an ice-water bath to stop the inactivation process, and the remaining PNPPase activity was assayed as described above.

### Effect of several compounds on the *p*-nitrophenylphosphatase activity

Reactions were carried out in 50 mM acetate buffer at pH 6.0, containing 1 mM of PNPP and the following compounds: phosphate (10 mM); EDTA (10 mM); arsenate (1 mM); magnesium (2 mM); calcium (1 mM); zinc (1 mM); cobalt (1 mM); levamisole (10 mM); sodium tartrate (10 mM); bafilomycin A1 (1 mM); oligomycin (1.5 mg/ml); ouabain (1.3 mM); pantoprazol (6 mM); PHMB (1 mM); and vanadate (0.5 mM), in a final volume of 1.0 ml. The reaction was initiated by the addition of the enzyme and stopped by the addition of 1.0 ml of 1.0 M NaOH at the appropriate time. In each determination standards were included to estimate the non-enzymatic hydrolysis of substrate.

### Effect of pH on *p*-nitrophenylphosphate hydrolysis by membrane-bound acid phosphatase

Assays were buffered with 50 mM acetate for the pH range 3.5-6.5, and 50 mM Tris–HCl for the pH range 6.5-8.0; each reaction contained 1 mM of PNPP. There was no significant difference among the two buffers used at pH 6.5. The pH before and after each kinetic determination did not differ by more than 0.05 units. The reaction was initiated by the addition of the membrane-bound enzyme and stopped with 1.0 ml of 1.0 M NaOH at the appropriate time. In each determination standards were included to estimate the non-enzymatic hydrolysis of substrate.

### Determination of protein concentrations

Protein concentrations were determined according to the method described by Hartree [19]. Bovine serum albumin was used as the standard in both cases.

### Estimation of kinetic parameters

V, v, K_0.5_ and n obtained from substrate hydrolysis reactions were fit using a microcomputer as described by Pizauro *et al.*[20]. Data are reported as the mean of triplicate determinations that differed by less than 5%.

## Results and discussion

**Figure 1:**
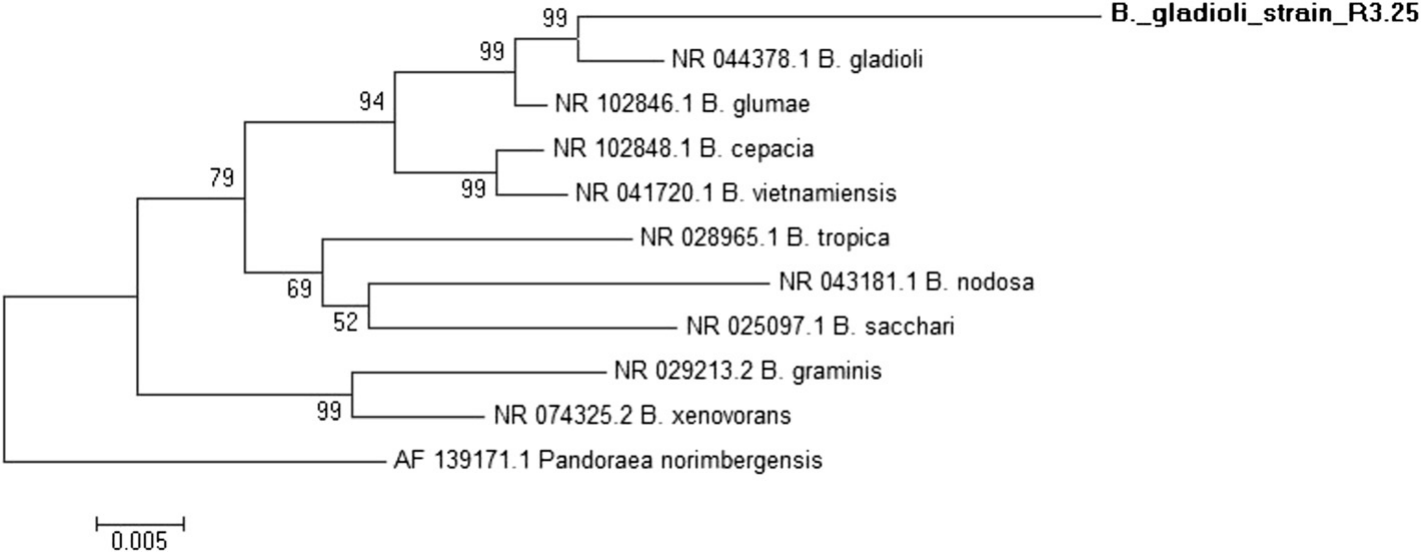
**Dendrogram of the partial sequences of 16S rDNA, proving the similarity of the isolated R 3.25 with *Burkholderia gladioli*.**

To quickly and reliably determine if the isolate belonged to the *Burkholderia* species, the amplified 16S rDNA was compared with the most similar found in databases, and phylogenetic analysis showed that the strain belonged to a *Burkholderia* species (Figure 1). This isolate clustered with a set of *Burkholderia* strains classified as *Burkholderia gladioli* (GenBank BankIt accession nº JN 700991).

**Figure 2:**
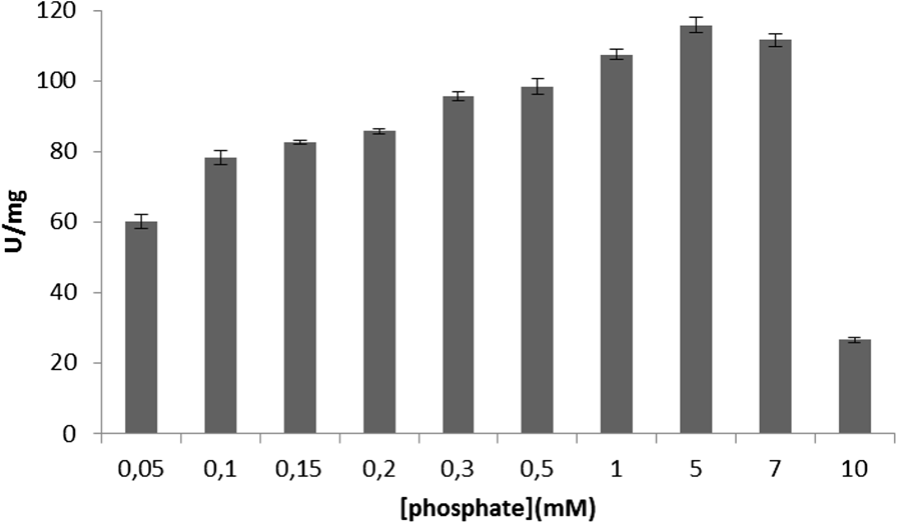
**Expression of PNPPase activity of membrane-bound acid phosphatase in *Burkholderia that was grown in medium containing increasing concentrations of phosphate.***

Figure 2 shows the effect of increasing concentrations of phosphate in the medium used to grow *Burkholderia gladioli.* The acid phosphatase activity was maximal at 5 mM phosphate, and reduced activity was observed when *Burkholderia gladioli* was grown in medium containing higher concentrations of phosphate. These results suggest that acid phosphatase in *Burkholderia gladioli* is synthesized exclusively under Pi-limiting conditions, which is characterized as Pi-repressible activity. The behaviors observed here show that acid phosphatase activity is downregulated by exogenous Pi concentration, suggesting that there is a tight coupling among the utilization of exogenous Pi, mobilization of endogenous reserves, and derepression of acid phosphatase. In fact, several phosphatases from plant and fungal origins were shown to be induced by phosphate deficiency [21-24].

The benefits to use differential centrifugation to obtain the membrane-bound acid phosphatase is that this method is easy to reproduce, fast and highly reproducible. This method resulted in the separation of two fractions. The supernatant containing soluble proteins, which represented less than 5% of total activity and the pellet, corresponding to the membrane-bound enzyme, which represented more than 95% of total activity, and showed specific activity of 103.9 U/mg for the chromogenic substrate; this fraction was used in further studies.

**Figure 3:**
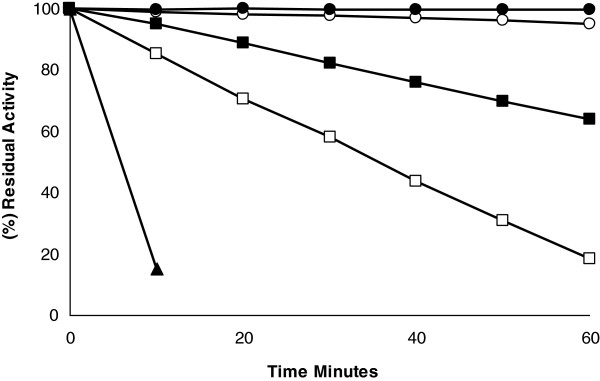
**Thermal inactivation of membrane-bound acid phosphatase.** The residual activity was determined by the addition of 50-μL aliquots to 50 mM acetate buffer, pH 6.0, containing 1 mM PNPP, in a final volume of 1.0 ml. Values are the mean of triplicates determinations that differed by less than 5% variation. (●) 50°C; (▲) 70°C; (■) 60°C; (□) 65°C; (○) 55°C. Inset: Arrhenius plot for the above data.

Membrane-bound enzyme was stable after 6 h of incubation at 45°C in 100 mM acetate buffer at pH 6.0, but the enzyme was inactivated at higher temperatures, exhibiting a t_1/2_ that varied from 23 h at 50°C to 5 min at 70°C. When the temperature was increased from 10°C to 70°C, enzyme inactivation followed first order kinetics, and no break in the inactivation curves was observed from 10°C to 25°C (Figure 3), suggesting that this enzyme is associated with lipids in the membrane but is not an intrinsic membrane-bound enzyme [25]. This behavior is consistent with that reported for alkaline phosphatase from rat bone matrix-induced cartilage [26], which is anchored to the membrane by phosphatidylinositol [27,28], and acid phosphatase with phytase activity from *Mucor hiemalis*[29]. In addition, according to Kondo *et al*. [30], acid phosphatase from *Burkholderia* is a glycoprotein that is translocated during glycosylation from the cytoplasm to the outer membrane and then excreted into the environment.

**Figure 4:**
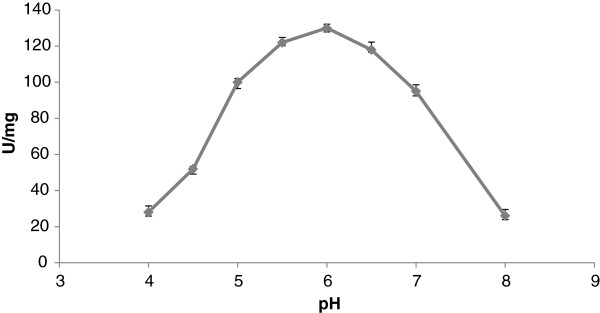
**pH sensitivity of the catalysis of membrane-bound acid phosphatase from *Burkholderia gladioli*.** Enzymatic assays containing 1 mM PNPP were buffered with 50 mM acetate for the pH range 3.5-6.5, and 50 mM Tris–HCl for the pH range 6.5-8.0. There was no significant difference between the two buffers used at pH 6.5. The pH before and after each determination did not differ by more than 0.05 units, and the reaction was initiated by the addition of membrane-bound enzyme.

The rate of hydrolysis of PNPP (Figure 4) by membrane-bound enzyme reached a maximum at pH 6.0, and this pH was selected as the incubation medium for the activity assays of acid phosphatase. This value for the optimum pH is similar to those reported for bacterial nonspecific acid phosphohydrolases [31], acid phosphatase from *Aspergillus ficuum*[21], and *Burkholderia cepacia*[32].

**Figure 5:**
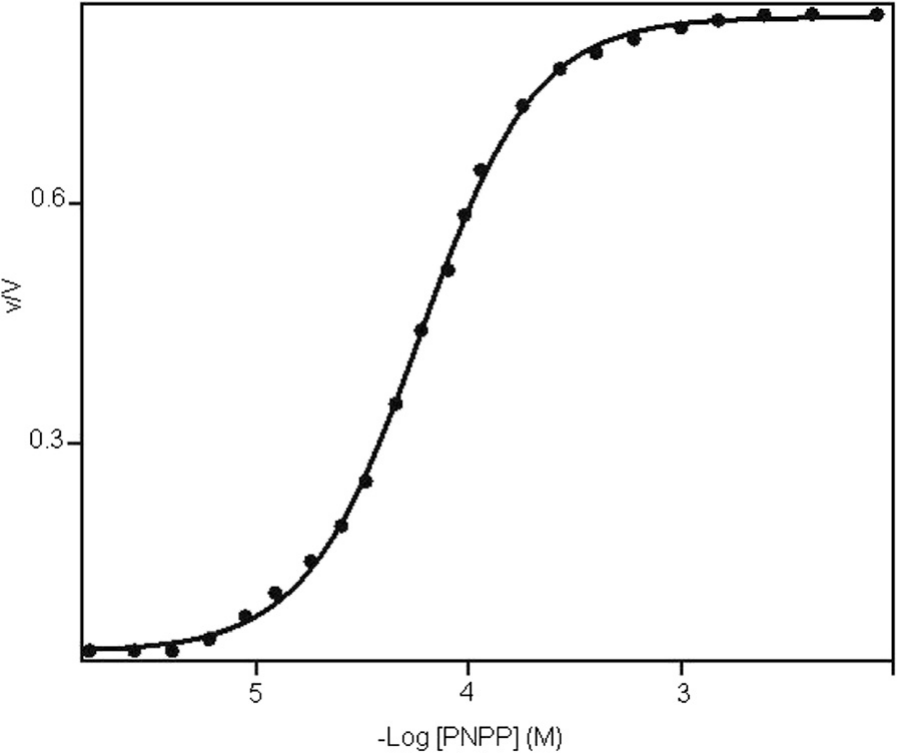
**Effect of substrate concentration on phosphohydrolytic activity of membrane-bound acid phosphatase from *Burkholderia gladioli*.** The PNPPase activity was assayed discontinuously at 37°C. Standard assessment conditions were 50 mM acetate buffer, pH 6.0, and containing increasing concentrations of substrate from 0.1 μM to 10 mM.

The hydrolysis of PNPP by the enzyme exhibited a hyperbolic relationship with increasing concentration of substrate and no inhibition by excess of substrate was observed. The specific activity of the enzyme for the hydrolysis of PNPP was 113.5 U/mg and K_0.5_ = 65 μM (Figure 5). Kinetic data revealed that the hydrolysis of PNPP exhibited cooperativity with n = 1.3.

The ability of this enzyme to dephosphorylate phosphoesteres and the observed magnitudes of the kinetic values are consistent with those obtained for acid phosphatase from other sources [33,31]. In addition, the enzymatic properties of acid phosphatase were virtually identical to the acid phosphatase of *E. histolytica*[34], which catalyzes *p*-nitrophenylphosphate hydrolysis under acid pH conditions.

The effects of several compounds on *p*-nitrophenylphosphatase activity of the enzyme are shown in Table 1. EDTA and tartrate reagents that were present in concentrations up to 10 mM showed only minor effects on enzymatic activity. The lack of inhibition by EDTA and sodium tartrate on PNPPase activity and the lack of dependence on magnesium, calcium, cobalt and zinc was also observed, suggesting that metal ions are not involved in maintaining enzyme activity [35,26,38]. The significant inhibition observed for vanadate (94%), does not imply, unequivocally, that this membrane-bound enzyme is a specific P-type ATPase. This interpretation is supported by the lack of inhibition by ouabain, which is an inhibitor of the Na^+^/K^+^-ATPase. Bafilomycin is also a highly specific inhibitor of vacuolar H^+^-ATPases (V-ATPase) type in animal cells, plant cells and microorganisms [20] and does not inhibit the enzyme studied here. In addition, vanadate ions form a trigonal bipyramidal transition state at the active site of phosphatase, which bears some resemblance to the metastable intermediate occurring during the hydrolysis of phosphate esters [39]. Phosphate and arsenate are well known as inhibitors of acid phosphatase activity; the inhibition of PNPPase activity by phosphate and the more efficient inhibition by sodium arsenate, which is its structural analog, suggest a common mode of binding to the active site [40]. Similar to other phosphohydrolases, membrane-bound acid phosphatase is significantly inhibited by sodium orthovanadate [41,42] and p-hydroxymercuribenzoate [43,44], which suggests that the sulfhydryl residue of cysteine is essential for its activity.

**Table 1:** Relative effectiveness of several reagents on the activity of membrane-bound acid phosphatase from the R 3.25 isolate

**Reagent**	**Residual activity**
Phosphate (10 mM)	38
EDTA (10 mM)	95
Arsenate (1 mM)	14
Magnesium (2 mM)	96
Calcium (1 mM)	94
Zinc (1 mM)	97
Cobalt (1 mM)	94
Levamisole (10 mM)	93
Sodium tartrate (10 mM)	95
Bafilomycin A1 (1 mM)	98
Oligomycin (1.5 mg/ml)	93
Ouabain (1.3 mM)	92
Pantoprazol (6 mM)	95
PHMB (1 mM)	5
Vanadate (0.5 mM)	6

Specific activity of 100% corresponds to 109.27 U/mg.

The main mechanism for mineral phosphate solubilization is the production of organic acids, and acid phosphatases play a major role in the mineralization of organic phosphorous in soil [45]. Considering that acid phosphatase is bound to the external membrane surface and therefore exposed to extracellular medium, our results bring an important insight on the mechanism of mineral phosphate solubilization by this bacterium and on plant nutrition through the increase in P uptake by the plant, mainly in soils with low levels of phosphate that are found in many regions of the world.

Although *Burkholderia gladioli* is known as a pathogen in some plant species [2] and causes opportunistic infection in severely immunocompromised humans [46], Bae [47] reported that a strain of this species have the ability to suppress pathologies caused by *Pythium ultimum*. In addition, *B. gladioli* has been described as a possible biofertilizer because of its capacity to fix nitrogen, mobilize phosphorus and stimulate plant growth [48-50]. It should be emphasized that *B. gladioli* also promotes beneficial effects as plant growth and nitrogen fixation in sugarcane crops [50]. Therefore, it may be possible to use this bacterium as a biofertilizer for specific crops, besides biochemical studies can contribute to elucidate its mechanisms.

## Conclusion

Through analysis of the 16S rDNA our strain was classified as *Burkholderia gladioli* (GenBank BankIt accession nº JN 700991), therefore, phylogenetically distant from the *Burkholderia cepacia complex* (Bcc species). The synthesis of membrane-bound non-specific acid phosphatase, strictly regulated by phosphate, and its properties suggest that this bacterium has a potential biotechnological application to solubilize phosphate in soils with low levels of this element for specific crops.

## Competing interests

The authors declare that they have no competing interests.

## Authors’ contributions

THR executed the enzymatic assays and enzyme extraction and analyzed the data, along with AMG and LFJS. EANP and EGML performed the genetic studies and analysis. JMPJ conceived of the study and wrote the manuscript. All authors read and approved the final manuscript.
